# The effect of a tele-educational intervention on modifying dysfunctional sexual beliefs of pregnant women: a randomized controlled trial

**DOI:** 10.1186/s12884-022-04773-1

**Published:** 2022-06-17

**Authors:** Shirin Khoddam, Razieh Lotfi, Kourosh Kabir, Effat Merghati- Khoei

**Affiliations:** 1 Student Research Committee, Alborz University of Medical Sciences, Karaj, Iran; 2 Department of Midwifery, School of Nursing and Midwifery, Alborz University of Medical Sciences, 1st Golestan- Eshteraki Boulevard, Baghestan, Karaj, Iran; 3 Social Determinants of Health Research Center, Alborz University of Medical Sciences, 1st Golestan- Eshteraki Boulevard, Baghestan, Karaj, Iran; 4 Department of Community Medicine, School of Medicine, Alborz University of Medical Sciences, Karaj, Iran; 5 Spinal Cord Injury Research Center (BASIR), Neuroscience Institution, Tehran University of Medical Sciences, Tehran, Iran

**Keywords:** Pregnancy, Sexuality, Tele-education, Text message, Dysfunctional sexual beliefs

## Abstract

**Background & aim:**

Some cultural scenarios in pregnancy and childbirth reinforce dysfunctional sexual beliefs that reverse changes in the couple's sexual life. The present study aimed to investigate the effect of education by sending text messages on modifying dysfunctional sexual beliefs in pregnant women.

**Methods & materials:**

This study is a randomized clinical trial, and 82 eligible pregnant women referred to educational-medical centers to receive prenatal care were randomly assigned to intervention or control group. The intervention group received 24 text messages during eight weeks (three text messages per week), and the control group received only routine care. Data was collected through a demographic questionnaire, reproductive profile, Spinner's Dyadic Adjustment Scale (DAS), and dysfunctional sexual beliefs questionnaire. Both groups completed the questionnaires before and one week after the intervention. Independent t-test, paired t-test, and analysis of covariance was used to analyze the data.

**Results:**

The findings revealed no statistically significant difference in the baseline Dyadic Adjustment mean scores of control (132.4 ± 11.01) and intervention (130.10 ± 10.66) groups. Paired t-test analysis showed that the mean score of dysfunctional sexual beliefs was significantly decreased from (29 ± 7.61) at baseline compared to one week after intervention (10.54 ± 6.97) (*p* < 0.001). Analysis of covariance test to compare the scores of dysfunctional sexual beliefs in the intervention group (10.54 ± 6.97) and control group (26.80 ± 7.80) showed a statistically significant difference (*p* < 0.01) with an effect size of 0.67.

**Conclusion:**

This study showed that sending text messages to mobile phones of pregnant women has corrected their dysfunctional sexual beliefs. Therefore, this approach can be used in pregnancy care to promote women's sexual health.

**Trial registration:**

Clinical trial registry: IRCT20161230031662N9.

**Supplementary Information:**

The online version contains supplementary material available at 10.1186/s12884-022-04773-1.

## Introduction

Pregnancy is one of the most sensitive periods in women's lives that can affect sexual relationships by creating physical and psychological changes [1, 2]. Sexual behavior is well defined by a biopsychosocial model [3] and is closely related to a woman's beliefs [4]. During pregnancy, sexual behaviors and attitudes are influenced by sexual value systems, indigenous culture, and traditional or religious beliefs [5]. Studies suggested changes in sexual behavior of pregnant women that are not related to sexual functioning but are associated with sexual distress [6]. Pregnant women's sexual behaviors alter due to many reasons such as; the fear of harm to the fetus, pain, miscarriage, discomfort, the belief that sex in pregnancy is a sin, fatigue, enlarged abdomen, nausea, and vomiting [7].

In addition, spouses of pregnant women sometimes come up with a decrease in their sexual desire and activity due to fear of harm to the fetus, the belief that sexual activity is immoral during pregnancy, and the stress of fatherhood [1]. While there are no restrictions on sexual activity in normal pregnant women [8], most couples are unaware of how to have sex during pregnancy. Despite the need for education on sexual issues during pregnancy, this is often ignored [9, 10]. Despite the high prevalence of sexual dysfunction and sexual beliefs about pregnancy [10, 11], few are consulted and treated [12]. Dysfunctional sexual beliefs endanger a couple's sexual life and influence their sexual function [13]. In many cases, the individual is so influenced by dysfunctional beliefs in sexual practice that clarifying and reconstructing them is an essential step toward creating desirable sexual relations between couples [6].

Despite the need for educational counseling interventions, there are gaps in the reproductive health system [12]. Studies have shown that face-to-face sex education can improve dysfunctional sexual beliefs and, consequently, the quality of sexual life, sexual satisfaction, and marital relationship of pregnant women [14, 15]. Still, it is not clear whether tele-education can have such an effect.

Telehealth is beneficial for easier access to health care [16], and some methods, such as text messaging, are considered effective and acceptable interventions for promoting health behaviors [17]. Delivering SMS text messages is a cost-effective strategy to provide maternal health care [18, 19]. It is associated with improvement in obstetric outcomes, perinatal smoking cessation, breastfeeding [20], self-care of pregnant women [21], and doctor-patient communication [22]. Moreover, mobile phone text-based interventions have received more attention in reproductive and sexual health studies worldwide [23, 24].

Interventions to promote the sexual health of pregnant women and modify their beliefs about sexuality in pregnancy need an effective model that could increase access to psycho-education materials. Delivering text message-based psycho-education can overcome some common barriers such as specialist availability, cost, and geographic access for people [25] and also help provide an effective intervention to increase individuals' mental and physical health [26]. A pilot study based on a 12-week intervention of text messaging could significantly affect decreasing depression symptoms [25]. Even in the critical era of the COVID-19 pandemic, tele-health education could help pregnant women lessen their pregnancy-related stress and anxiety [27]. The present study aimed to determine the effect of tele-education on modifying the dysfunctional sexual beliefs of pregnant women.

## Materials and methods

### Design

This study is a randomized clinical controlled trial conducted from July to November 2019 on 82 pregnant women aged 18–45 with 14 to 31 weeks gestational age based on LMP or ultrasound records. Samples of the study were expectant mothers referred to Kamali and Imam Ali educational medical centers for receiving prenatal care. These two centers are specialized academic hospitals of Alborz University of Medical Sciences, including an active gynecology clinic and access to research samples. The participants were recruited by convenience sampling and then randomly assigned to the intervention or control group. Block randomization was done by the online randomization site "Sealed envelope website" using the block size of four. A colleague out of the study conducted randomization and concealment. Both groups completed the demographic questionnaire, reproductive profile, dysfunctional sexual beliefs scale, and Spinner's Dyadic Adjustment Scale (DAS). Individuals who obtained scores less than 100 from DAS questionnaire were excluded from the study. Then, one week after the intervention, both groups completed a dysfunctional sexual belief questionnaire. The intervention group was asked an item about satisfaction with a 5-point Likert scale from "completely satisfied" to "completely dissatisfied". At the end of the intervention, an educational face-to-face session was presented for the control group.

### Participants

The participants fulfilled the inclusion criteria, including the age range of 18 to 45 years, gestational age of 14–31 weeks, Iranian nationality, intended pregnancy, monogamous relationships, being physically healthy, single pregnancy, no addiction in couples, having a personal cell phone, and marital adjustment. The study exclusion criteria were: self-reported mental illness, sexual dysfunction or any known psychiatric disorder in the couples, high-risk pregnancy such as vaginal bleeding, placenta previa, assisted reproductive techniques, threatened abortion, chronic medical conditions, previous history of miscarriage or intrauterine death of the fetus.

### Sampling

**Fig.1:**
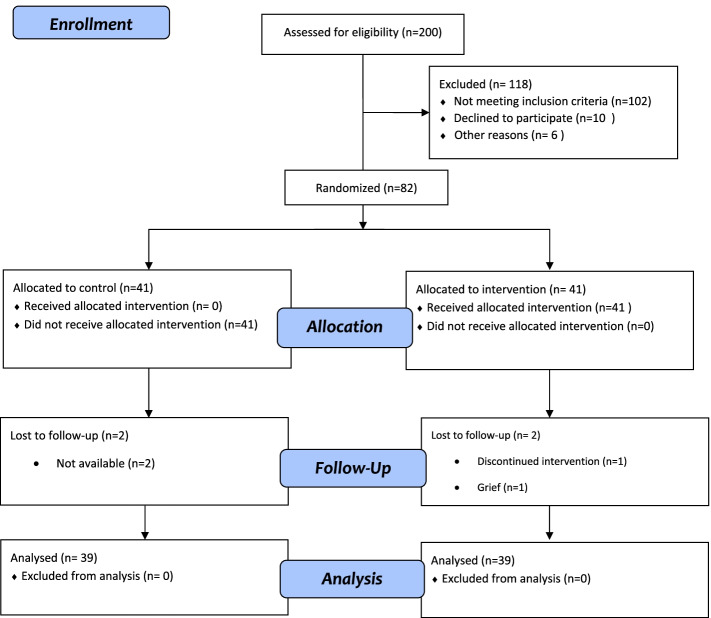
2010 CONSORT flow diagram

During three months, 200 pregnant mothers were registered and evaluated in the prenatal clinic by attending the centers every other week and interviewing mothers. First of all, the participants were explained the aim of the study and then included in the study after taking the informed written consent if they met the inclusion criteria. The participants referred to the prenatal clinics of two educational hospitals were recruited by convenience sampling. Forty-one participants were allocated to each group by block randomization. The first researcher prepared 21 opaque envelopes (to obscure the envelope's contents), and each random sequence created was recorded on a card and placed inside the envelope. They were then placed in a larger envelope. One of the colleagues outside the research team was asked to keep the envelopes. Finally, 82 mothers were included in the intervention. Figure 1 presents the flowchart of the study.

The number of samples using G * Power software version 3.1.2 was estimated to be 34 in each group according to α = 0.05, effect size = 0.7, and β = 0.20. The attrition rate was considered equal to 20 percent, and then the total sample size was 82 (41 in each group). This study was registered in Alborz University of Medical Sciences with the code of ethics IR.ABZUMS.REC.1398.024 and registered in the Iranian Clinical Trial database with the code IRCT20161230031662N9. The first registration was done on 15/07/2019.

### Intervention

The SMS content in this study was designed based on a literature review. Training messages were sent to pregnant women at the gestational age of 14 to 31 weeks in the intervention group for eight weeks; one message every other day, at 10 AM. The educational messages were related to the anatomy of the female reproductive system, uterus, fetus, and amniotic sac. Moreover, it was clarified that there is no relation between intercourse and miscarriage or rupture of the amniotic sac or the onset of childbirth pains in normal pregnancies. We have also provided educational messages regarding appropriate sexual positions during pregnancy, the use of condoms to prevent the transfer of infection, physiological changes during pregnancy, body image of the pregnant women to strengthen the mother's self-confidence, and modifying beliefs about guilt and immorality of sex during pregnancy.

The participants were suggested to write the received messages in a separate notebook and review them repeatedly with their spouses. A multiple-choice test was sent to participants in the middle of the training course to ensure they noticed the messages. An in-person training session was held for the control group at the end of the study.

Before delivering any message, we asked a question. Samples of questions and messages were: "Can the sexual intercourse during pregnancy be considered a "sin" according to Sharia?" Then the message was:

"The pregnant mother should know that having sex during pregnancy is completely normal, and she does not commit any sin or mistake. The goal is to reach a point of peace of mind and meet the couple's emotional needs. Sex and sexual intercourse do not only mean satisfying sexual desire but also hugging, kissing, and expressing love causes more intimacy and maintains family unity."

"Or another question and message were: "Does sex during pregnancy cause a ruptured amniotic sac?" Then the message was: "During sexual intercourse, the penis enters the vagina but has no contact with the amniotic sac around the fetus, so if sex is not very deep in the last trimester, it has no significant association with premature rupture of the sac."

### Measurements

This study's data collection tools include demographic and individual characteristics, a fertility profile questionnaire, the Sexual Dysfunctional Beliefs Questionnaire, and Dyadic Adjustment Scale (DAS). Also, one question about satisfaction with the training was formulated on a five-point Likert scale, from completely satisfied to completely dissatisfied, which was asked at the end of the intervention using SMS by the intervention group. The primary variable was dysfunctional sexual beliefs.

#### Demographic and individual characteristics

Demographic and individual characteristics: included variables such as age, education, occupation of the couple, and age of marriage.

Fertility profile questionnaire: Number of pregnancies, number of abortions, number of live children, gestational age, intercourse method, and number of intercourses per week and month were assessed in this questionnaire.

#### Questionnaire of dysfunctional sexual beliefs

The questionnaire on dysfunctional sexual beliefs in pregnant women, with an extensive literature review and based on the study of Sosa et al. [28–30], was designed by the research team, and the validity and reliability were evaluated. The final form of this questionnaire has 13 questions, and the scoring method is based on a five-point Likert scale, from "strongly disagree" to "strongly agree," and the scores were from 0 to 4. A higher score indicates more dysfunctional beliefs.

Qualitative and quantitative content validity methods assessed the validity of this questionnaire. The initial questionnaire consisting of 21 items was reviewed by ten specialists in reproductive health, obstetrics, psychology, sex therapy, and health education and was studied based on corrective opinions. Quantitative content validity was assessed by two indicators, CVR and CVI. In examining the content validity ratio, the standard index for ten experts was 0.62 based on the Lawshe table [31]. Accordingly, eight items were removed, and 13 out of 21 remained. The reliability of the dysfunctional sexual beliefs questionnaire was confirmed with Cronbach's alpha = 0.89.

#### Dyadic Adjustment Scale (DAS)

Dyadic adjustment scale (DAS) has 32 items and four dimensions: marital satisfaction, marital cohesion, marital agreement, and expression of love. The score range of this questionnaire is from zero to 150, and a higher score is a sign of more adjustment. On this scale, a score of 100 or more means adjustment of individuals, and scores less than 100 tell marital problems. Scoring is based on a 5-point Likert scale. High scores indicate higher marital quality. The validity and reliability of this scale have been approved in Iran [32]. In the present study, the reliability of this scale was determined to be 0.91 based on Cronbach's alpha.

## Results

The demographic characteristics of the participants are given in Table 1. There were 39 subjects in each intervention and control group. There was no statistically significant difference between the intervention and control groups regarding demographic variables. The mean and standard deviation of the scores of the marital adjustment for the control and intervention groups before the intervention show that the mean scores of marital adjustment in the control and intervention groups were 132.41 and 130.10, respectively, and according to t = -0.940, at 95% confidence level, marital adjustment was not significantly different.

**Table 1:** Statistical description of demographic and fertility information by the control and intervention groups

Variable	Control group		Intervention group		Statistics	
Age	*N* = 39	percent	*N* = 39	percent	*χ*.^2^	*P* value
18–27	25	64.1	17	43.6	3.342	0.188
28–37	13	33.3	20	51.3		
38–45	1	2.6	2	5.1		
Education						
Under Diploma	10	25.6	4	10.3	5.781	0.216
Diploma	20	51.3	23	59		
Higher Education	9	23.1	12	30.8		
Employment						
Yes	0	0	2	5.2	2.053	0.358
No	39	100	37	94.9		
Marriage age						
Less than 20	13	33.3	9	23.1	1.024	0.599
20–30	25	64.1	29	74.4		
31–45	1	2.6	1	2.6		
Spouse age						
18–27	10	25.6	9	23.1	2.053	0.358
28–37	26	66.7	23	59		
38–45	3	7.7	7	17.9		
Spouse education						
Under Diploma	11	28.2	9	23.1	2.610	0.625
Diploma	18	46.2	23	59		
Higher Education	10	25.7	7	18		
Gravida						
1	23	59	20	51.3	1.849	0.397
2	13	33.3	12	30.8		
3 and more	3	7.7	7	17.9		
Gestational age						
14–18	11	28.2	6	15.4	2.418	0.490
19–23	13	33.3	13	33.3		
24–27	7	17.9	11	28.2		
28–31	8	20.5	9	23.1		
Number of intercourses (last month)						
0	6	15	7	18	1.468	0.832
1	20	51	19	49		
2	10	26	8	20		
3	3	8	4	10		
4	0	0	1	3		
Method of intercourse						
Vaginal	27	69.2	29	74.4	0.916	0.822
Oral	1	2.6	2	5.1		
Combinational	5	12.8	4	10.3		
None	6	15.4	4	10.3		

Paired t-test showed that the difference between the mean scores of the dysfunctional sexual beliefs in the control group was not significant (*P* = 0.096). Still, it was significant in the intervention group (*P* < 0.01). (Table 2).

**Table 2:** Dysfunctional sexual beliefs scores in the two groups before and after the intervention

Variable	Group	Stage	Mean ± Standard deviation	paired t-test	*p*-value
Dysfunctional sexual beliefs	Control	Before intervention	25 ± 6.59	1.708-	0.096
		After intervention	26.80 ± 7.80		
	Intervention	Before intervention	29 ± 7.61	15.237	0.001
		After intervention	10.54 ± 6.97		

The homogeneity analysis of the regression line slope as a default covariance analysis showed that the significance level of the mutual effect line of the group before the intervention (*p* = 0.177) was greater than 0.05. Therefore, the regression homogeneity hypothesis is accepted.

The significance level of Levene test was sig = 0.76, which indicates the equality of variances between the two groups (Table 3). Therefore, it can be concluded that there is a significant difference between the mean scores of the post-test variable of dysfunctional sexual beliefs in the intervention and control groups.

**Table 3:** The difference between the mean scores of dysfunctional sexual beliefs in the intervention and control groups after the intervention

		Levene test for equality of variances		The dependent t-test for means						
		F	sig	t	df	Sig (2-tailed)	Mean difference	SD difference	95% Confidence Interval of the Difference	
									High	Low
Dysfunctional sexual beliefs (post-test)	Assumption of unequal variance	38.0	0.76	-9.70	76	0.00	-16.25	67.1	-19.59	-12.92
	Assumption of unequal variance			-9.70	75.05	0.00	-16.25	67.1	-19.59	-12.91

The results of analysis of covariance to compare the scores of dysfunctional sexual beliefs in the intervention and control groups after the intervention showed that the value of F is equal to 152.52, and its significance level is less than 0.01 (*p* < 0.01) (Table 4). Based on this and considering the lower average scores of the intervention group after the intervention, it can be concluded that text messages have been effective and could correct the sexual beliefs of pregnant women. The effect of text messages on correcting sexual beliefs was 67%.

**Table 4:** Results of analysis of covariance to compare dysfunctional sexual beliefs in the intervention and the control groups

Source of changes	Sum of squares	Degree of freedom	Average squares	*F* value	Significant level	Effect size
Pre-test	1143.3	1	1143.3	28.44	0.01	0.27
Group	6131	1	6131	152.52	0.01	0.67
Error	3014.8	75	40.19			
Total	9311.3	77				

## Discussion

This study showed that text messages effectively corrected the sexual beliefs of pregnant women. Education by text message on sex in pregnant women is being studied for the first time in Iran. Today, mobile phones can help improve and strengthen preventive health care in low-income and middle-income countries. The use of text messages, even only as a reminder, will still promote health.

The prevalence of mobile phones, especially in Iran, has reached about 90%. This issue leads to all people receiving the messages despite their busy schedules. For this reason, we could expect changes through text messages in pregnant women [33]. Using mobile phones in educational programs through text messages for pregnant women improves access to education, reduces social costs, promotes justice in education, and optimizes time [18, 19].

Due to misconceptions and changes in pregnant woman's body image, reduced feeling of attractiveness to the husband, fear of harm to the fetus, and risk of abortion [34], many studies have shown that health behaviors of pregnant women can be modified and corrected through tele-education [14, 33, 35]. Text messaging can increase a large population's reproductive health knowledge and reduce the risks of unwanted pregnancies in female adolescents [36]. It seems that integrating this mode of education in primary health services is necessary. Tele-education can reduce the dysfunctional beliefs of pregnant women remotely and without the need for physical presence, which can be a practical approach to achieving sexual health. A large part of the feeling of guilt may originate from this belief that coitus may be harmful to the fetus.

Alizadeh et al. showed that more than half of pregnant women had been worried about the possibility of harm to the fetus during sexual activity before the educational intervention. After a one-hour one-on-one individual session, this rate was reduced to 3.3% in the intervention group and remained at 50% in the control group [29]. It shows the effectiveness and necessity of training to correct sexual beliefs, aligning with the present study results.

A study showed a significant decrease in the score of dysfunctional sexual beliefs related to the harms of intercourse to the fetus, such as abortion, infection, rupture of the sac, premature delivery, etc., which is in line with the present study. But the score of a woman's guilt feelings about sex during pregnancy and the perception that they are unattractive to their husbands and that sex was immoral during this period did not change significantly from their and their husbands' point of view [30]. The guilty feelings about the intercourse are started from early pregnancy, and they may need a longer time to be relieved. One difference between our study and the above-mentioned study was that our intervention was done over a more extended time, emphasizing re-reading the messages together with the spouse, which caused a significant reduction in scores of all 13 dysfunctional sexual beliefs items. In the present study, the belief in guilt caused by sexual intercourse in pregnancy was sharply reduced with training in the intervention group. Still, the control group, who did not receive SMS, increased significantly with gestational age. This finding was contrary to the results of a study that could not change the mother's guilt [29].

The importance of correcting sexual beliefs in pregnant women is the effect that incorrect sexual beliefs have on the quality of sexual life and other aspects of sexuality. Studies have shown the association between women's sex knowledge and sexual behaviors at childbearing age [29]. Fear of harm to the fetus, fear of miscarriage or premature birth, and fear of infection are present in more than half of women [29, 30]. More than 65% of the partners of pregnant women do not have the desire to have intercourse in pregnancy because they fear harm to the fetus [12]. The present study also showed that among the study participants, the highest score of dysfunctional belief in the pre-test of the intervention group was 43.6%. The pre-test of the control group was 38.5%, which was related to the husband's fear of harming the fetus during intercourse, which decreased by 10.3% in the post-test intervention group but increased by 46.6% in the control group.

Using questionnaires has limitations, including that individuals may not reflect the truth about questions for various reasons. However, the authors did proper communication and tried to justify the importance of accurate data. Therefore, the accuracy of the participants' statements was trusted. Only female participants and pregnant women at the 14 to 31 weeks of pregnancy were recruited for this study, which is a limitation. Although we tried to control some confounding variables, psychological variables and the mental health of individuals who may influence sexual beliefs have not been examined in this study. Still, we have controlled dyadic adjustment of the couples, which may be considered a strength of this study.

## Conclusions

Tele-education effectively modifies dysfunctional sexual beliefs of pregnant women in the second trimester of pregnancy. To promote pregnant women's sexual health, designing and applying this approach in routine prenatal care can play an essential role.

## Supplementary Information

**Additional file 1.**

## Data Availability

The datasets used and analyzed during the current study are available from the corresponding author on reasonable request.
