# Supplementary material for: The effect of a tele-educational intervention on modifying dysfunctional sexual beliefs of pregnant women: a randomized controlled trial
Source: BMC Pregnancy Childbirth. 2022 Jun 17;22:495. doi: 10.1186/s12884-022-04773-1 (PMC9204371; doi:10.1186/s12884-022-04773-1)
Supplement: Supplementary file 1 — Additional file 1. [file 12884_2022_4773_MOESM1_ESM.docx]

1. Is having sex and intercourse a problem during pregnancy?

Sexual intercourse is not a problem during pregnancy, and it does not harm you or your fetus, whether it is a boy or a girl. Your baby is protected by a double-walled water bag that is very durable and the uterine muscles surrounding it.

1. The cervical opening, which is located at the end of the vaginal canal, is completely closed before labor begins and protects the amniotic sac and the fetus.
2. Does sex during pregnancy lead to abnormalities and malformations of the fetus?

Intercourse during pregnancy will never cause the fetus to be abnormal. The cause of fetal abnormalities is hereditary and genetic or related to the effects of certain drugs, chemicals and microbial contamination.

1. Does intercourse cause abortion?

Sex and intercourse during pregnancy do not cause abortion; The cause of abortion is improper fetal development or other biological factors and has nothing to do with sex.

1. Does sex during pregnancy cause infection of the mother or fetus?

Inflammatory agents and infections cannot penetrate the healthy membranes of the fetus. If all the necessary tests for diagnosis of HIV, hepatitis, syphilis, gonorrhea, and other sexually transmitted infections are negative, intercourse may not lead to water bag infection.

1. Should condoms be used for sexual intercourse during pregnancy?

The use of condoms during pregnancy is only necessary if: you or your partner have a sexually transmitted infection, direct contact with these infections causes serious problems for the fetus, or you are unaware that you and your partner are not infected.

1. What is the role of the amniotic sac in pregnancy?

The amniotic sac is a pair of transparent and thin membranes that, as a strong physical shield, protect the fetus to a large extent from the pressures of the abdomen or trauma shortly before birth. Therefore, intercourse or sexual practice does not cause harm to the fetal head.

1. The fetus is floating in the amniotic sac, causing a sudden, sudden blow to the uterus, causing the fetus to descend into the amniotic sac and then return to its original position.
2. Does sex during pregnancy cause a ruptured amniotic sac?

During sexual intercourse, the penis enters the vagina. Still, it has no contact with the amniotic sac around the fetus. If sex is not very deep in the last trimester, it has no significant association with premature rupture of the sac.

1. Can sexual intercourse during pregnancy be considered a "sin" according to Sharia?

The pregnant mother should know that having sex during pregnancy is completely normal, and she does not commit any sin or mistake. The goal is to reach a point of peace of mind and meet the couple's emotional needs. Sex and sexual intercourse do not only mean satisfying sexual desire but also hugging, kissing, and expressing love causes more intimacy and maintains family unity.

1. Your fetus may move more after orgasm due to an increase in your heart rate and more heart output. It has nothing to do with the fetus feeling pain from your relationship or understanding your sex practice. So, do not feel guilty.
2. Does the fetus see an embryo during its life, or does it have cognitive powers?

By the end of the eighth month, the fetus's pupil is open and responds to light, but the fetus's visual power and cognitive network have not yet formed in utero, and the fetus has not the ability to capture images and memories in utero.

1. How is your sexual desire?

You may or may not want to have sex in the first trimester of pregnancy because of nausea and vomiting or feeling tired, which is perfectly normal. In the second trimester the hormones are more stable, and you are more sexually active and have better sexual desire. While in the third trimester, your weight and the shape of your abdomen change the status of intercourse for you and your spouse, and of course, by reading the message related to the positions, you can still have a successful relationship.

1. How does pregnancy affect my libido?

Sexual desire does not decrease during pregnancy, but pregnancy can cause a change in your sexual desire and comfort level, because, in each different trimester, hormones are released more or less and sexual intercourse. In inappropriate and unhealthy conditions, it gradually reduces sexual desire.

1. How many times should I have sex during pregnancy?

It should be done according to the desire and needs of the couple, and if there is spotting early in the pregnancy or in case the couple is worried about the safety of sexual intercourse, they can replace the non-penetration sex instead of intercourse.

1. Is intercourse painful in the second trimester?

In the second trimester, the size of the abdomen is still not very large and the increase in vaginal discharge makes the vagina more slippery and easier to have sex.

1. What is the best position for sex during pregnancy?

As long as you feel comfortable, most sexual situations are good during pregnancy. As the pregnancy progresses, try ways to get the best position with your creativity while maintaining mutual pleasure and comfort.

1. After four months, the traditional position should be changed, and it means that the woman does not lie on her back and the man on top. In such a situation, the pressure on the main blood vessels to the placenta and fetus will not be recommended, and it is better to have a side-to-side position.
2. Should I control my orgasm during sex?

Contractions due to female orgasm in pregnancy are very weak and do not cause miscarriage or premature birth, and reaching orgasm for many pregnant women may cause physical and mental relaxation and more intimacy.

1. Near the onset of labor, spontaneous contractions of the uterine muscles occur regularly and become perceptible to the mother. These contractions do not interfere with intercourse, and sex and orgasm will not cause premature labor.
2. Are husbands also afraid of having sex during pregnancy?

Some husbands avoid sexual intercourse for fear of harming the fetus or the mother's internal organs. They should know that a woman's vagina is a sexual organ 10-12 cm long and ends in the cervix. The cervix is a cylindrical muscle tissue, surrounded by internal and external holes completely closed before labor. The penis in the vagina does not put pressure on the fetus.

1. Couples should talk about the natural changes in the body during pregnancy and discuss sexual issues openly. If the mother is uncomfortable having sex during pregnancy, she should talk about these feelings with her husband and midwife.
2. Do the physical changes caused by pregnancy upset you?

During the second and third trimesters, you will experience physical changes such as the size of the abdomen and breasts, overweight, skin discoloration, brown spots on the face and abdomen, etc., which are completely normal, and then they usually go away with childbirth. You can learn ways to reduce complications and how to deal with them with your midwife's guidance.

1. When should we not have sex?

Excessive stimulation and manipulation of the breasts and semen that contain a prostaglandin hormone cause uterine muscle contractions. Therefore, talk to your midwife if you have any of the following you should avoid sexual intercourse: Unexplained bleeding from the vagina, amniotic fluid leakage, Insufficiency of the cervix, placental problems such as placental previa, history of preterm delivery, history of recurrent miscarriage, multiplication.
